# Automatic reconstruction of the delivered dose of the day using MR-linac treatment log files and online MR imaging

**DOI:** 10.1016/j.radonc.2019.12.010

**Published:** 2020-04

**Authors:** Martin J. Menten, Jonathan K. Mohajer, Rahul Nilawar, Jenny Bertholet, Alex Dunlop, Angela U. Pathmanathan, Michel Moreau, Spencer Marshall, Andreas Wetscherek, Simeon Nill, Alison C. Tree, Uwe Oelfke

**Affiliations:** a Joint Department of Physics at The Institute of Cancer Research and The Royal Marsden NHS Foundation Trust, London, UK; b Elekta AB, Stockholm, Sweden

**Keywords:** MR-linac, MR-guided radiotherapy, Intrafractional motion, Dose reconstruction

## Abstract

**Background and purpose:**

Anatomical changes during external beam radiotherapy prevent the accurate delivery of the intended dose distribution. Resolving the delivered dose, which is currently unknown, is crucial to link radiotherapy doses to clinical outcomes and ultimately improve the standard of care.

**Material and methods:**

In this study, we present a dose reconstruction workflow based on data routinely acquired during MR-guided radiotherapy. It employs 3D MR images, 2D cine MR images and treatment machine log files to calculate the delivered dose taking intrafractional motion into account. The developed pipeline was used to measure anatomical changes and assess their dosimetric impact in 89 prostate radiotherapy fractions delivered with a 1.5 T MR-linac at our institute.

**Results:**

Over the course of radiation delivery, the CTV shifted 0.6 mm ± 2.1 mm posteriorly and 1.3 mm ± 1.5 mm inferiorly. When extrapolating the dose changes in each case to 20 fractions, the mean clinical target volume $D_{98\%}$ and clinical target volume $D_{50\%}$ dose-volume metrics decreased by 1.1 Gy ± 1.6 Gy and 0.1 Gy ± 0.2 Gy, respectively. Bladder $D_{3\%}$ did not change (0.0 Gy ± 1.2 Gy), while rectum $D_{3\%}$ decreased by 1.0 Gy ± 2.0 Gy. Although anatomical changes and their dosimetric impact were small in the majority of cases, large intrafractional motion caused the delivered dose to substantially deviate from the intended plan in some fractions.

**Conclusions:**

The presented end-to-end workflow is able to reliably, non-invasively and automatically reconstruct the delivered prostate radiotherapy dose by processing MR-linac treatment log files and online MR images. In the future, we envision this workflow to be adapted to other cancer sites and ultimately to enter widespread clinical use.

## Highlights

• Novel dose reconstruction workflow based on log files and online MR imaging.
• Intrafractional motion and dose changes were clinically meaningless in most fractions.
• Rarely, large shifts caused the delivered dose to deviate from the treatment plan.
• Blueprint for dose reconstruction on MR-linacs for other cancer sites.

The clinical workflow to deliver external beam radiotherapy begins with the acquisition of anatomical and functional images of the patient [1]. Based on this information a treatment plan is designed, aiming to deliver a lethal amount of radiation to the tumor while sparing nearby organs-at-risk. In reality, the intended dose distribution is never delivered and the actually administered dose is unknown as the patient’s anatomy changes over the course of treatment. Tumor and organ movement, caused by respiratory, cardiac and gastrointestinal activity, result in underdosage of the tumor and unwanted exposure of healthy organs-at-risk [2], [3], [4]. Measuring anatomical changes and their impact on the delivered dose is crucial to accurately link the radiation dose distribution to the treatment’s therapeutic effect [5]. Improving this understanding is necessary to further optimize radiotherapy deliveries, inform patient stratification and ultimately drive the development and implementation of real-time adaptive radiotherapy techniques.

Radiotherapy treatment machines with integrated MR imaging are able to continuously survey the patient undergoing treatment without the need for implanted markers or additional imaging dose [6], [7], [8], [9]. They facilitate adaptation of the treatment to interfractional anatomical changes based on MR images acquired before the delivery of each fraction [10], [11]. On-board MR imaging can also be deployed to monitor intrafractional tumor motion and adapt the treatment in real-time using treatment beam gating [12], [13], [14], [15]. However, we are not aware of any studies reporting the use of online MR imaging to determine the delivered dose while accounting for intrafractional anatomical changes. This study presents a workflow that processes 3D MR images, 2D cine MR images and MR-linac machine log files to reconstruct the delivered dose. We measured intrafractional motion of the prostate and reconstructed the dose of the day in 89 prostate radiotherapy fractions administered with the Elekta Unity 1.5 T MR-linac (Elekta AB, Stockholm, Sweden) at our institute.

## Material and methods

### Clinical MR-guided prostate radiotherapy protocol

At our institute, prostate cancer patients are treated with the Unity 1.5 T MR-linac as part of the PRISM trial [16]. We aim to deliver 60 Gy in 20 fractions to the primary clinical target volume (CTV), comprising the entire prostate and the adjacent 1 cm of the seminal vesicles. Simultaneously, a secondary CTV, defined as the 2 cm of the seminal vesicles proximal to the primary CTV, is treated to 48.6 Gy. Additional planning constraints can be found in the Supplementary material.

The original fully optimized seven-beam intensity modulated radiotherapy (IMRT) treatment plan for the Unity MR-linac is generated on a pre-treatment CT image using the Elekta Monaco treatment planning system, version 5.40.00. At each fraction, the patient is positioned on the MR-linac treatment couch and a 3D T2-weighted MR planning image is acquired (sequence parameters in the Supplementary material). All contours are mapped from the pre-treatment image to the MR image via deformable image registration. An expert physician corrects the propagated CTV and critical organ-at-risk contours as needed. In order to calculate the dose on the MR image, bulk electron densities are assigned to the volumes defined by the contours [17]. The primary planning target volume (PTV) is created by expanding the primary CTV isotropically by 5 mm, except in posterior direction, where a 3 mm margin is added. The secondary PTV is generated by expanding the union of both CTVs by 5 mm.

Based on the updated contours, Monaco re-optimizes the treatment plan using the optimization constraints derived for the original treatment plan (“adapt-to-shape”) [18]. After contouring and re-planning, a 3D MR *verification image* is acquired. If the prostate has moved outside of the PTV during the previous steps, the treatment plan is adapted once more using the segment aperture morphing algorithm (“adapt-to-position”) [19].

During dose delivery, the patient is continuously monitored using a T2/T1-weighted balanced steady-state free precession MR sequence (parameters in the Supplementary material). The sequence images three orthogonal planes in cyclic order (transversal then sagittal then coronal) with an update frequency of 1.63 Hz for each plane. The acquired sagittal, coronal and transversal imaging planes intersect at the center of the target’s bounding box (defined as the smallest rectangular prism that encompasses the PTV while being aligned with the main patient axes). Additionally, the MR-linac dose delivery status is recorded every 40 ms in a treatment machine log file.

### Measurement of intrafractional prostate motion

In the context of the described workflow, we defined intrafractional motion as any anatomical changes occurring after the verification image has been obtained and the final treatment plan has been approved. In order to quantify the intrafractional motion, we were presented with two distinct image processing tasks: 1) determining the anatomical changes occurring between the acquisition of the 3D verification image and the beginning of 2D cine MR imaging and 2) monitoring the motion during radiation delivery while we survey the patient via 2D cine MR imaging.

For this study, we aimed at detecting shifts of the CTV in anterior-posterior and superior-inferior direction. Note that we disregarded shifts in left-right direction as well as any rotations and deformations of the prostate. Previous studies have indicated that these anatomical changes are comparably small and less impactful on the delivered dose [20]. Consequently, we discarded the coronal and transversal 2D cine MR images. The sagittal 2D cine MR images were affected by saturation band artifacts, manifesting themselves as dark stripes at the intersection of the imaging planes [21]. In order to mitigate the influence of these artifacts on the following image processing steps, we used a heuristic intensity correction [22]. We characterized the signal loss profile by acquiring MR images of a water tank using the same 2D cine MR sequence. The signal in the affected regions of the patient images was multiplied by the inverse signal loss profile. Furthermore, the first frame of each 2D cine MR sequence was discarded as this image is darker than the remaining images.

In order to determine the prostate shift between acquisition of the 3D verification image and the first 2D cine MR image, the images had to be further pre-processed. The 3D verification image was tri-linearly interpolated to match the position and resolution of the 2D MR images (see previous section and Supplemental material). Afterwards, the shift of the CTV was determined using a template matching algorithm [23]. A rectangular template was automatically extracted from the interpolated verification image by growing the bounding box of the CTV by 20 mm. The template matching algorithm then iteratively calculated the similarity between the template and a section of the 2D cine MR image. The search region of the 2D cine MR image encompassed the original CTV position as well as 25 mm in each direction and was sampled with a grid mandated by the image pixel size. Mutual information was used as similarity metric as it is robust against the different imaging contrasts of the T2-weighted 3D MR image and T2/T1-weighted 2D cine images [24]. The position yielding the highest similarity was deemed the current CTV position at the beginning of 2D cine MR imaging.

The motion trajectory during 2D cine MR imaging was also measured using a template matching algorithm. The first 2D image of the sequence was used to create a rectangular template by expanding the bounding box of the CTV by 6 mm. In order to find the CTV position in the subsequent frames, we defined a search region with a margin of 20 mm and used normalized cross correlation as similarity metric [25].

The position of the prostate at each time point during dose delivery was calculated by adding the determined CTV shift between acquisition of the verification image and 2D cine MR imaging to the continuous motion trajectory detected during 2D cine MR imaging. In all cases, we visually assessed the performance of the image processing algorithms to rule out gross failures.

### Dose reconstruction workflow

**Fig. 1:**
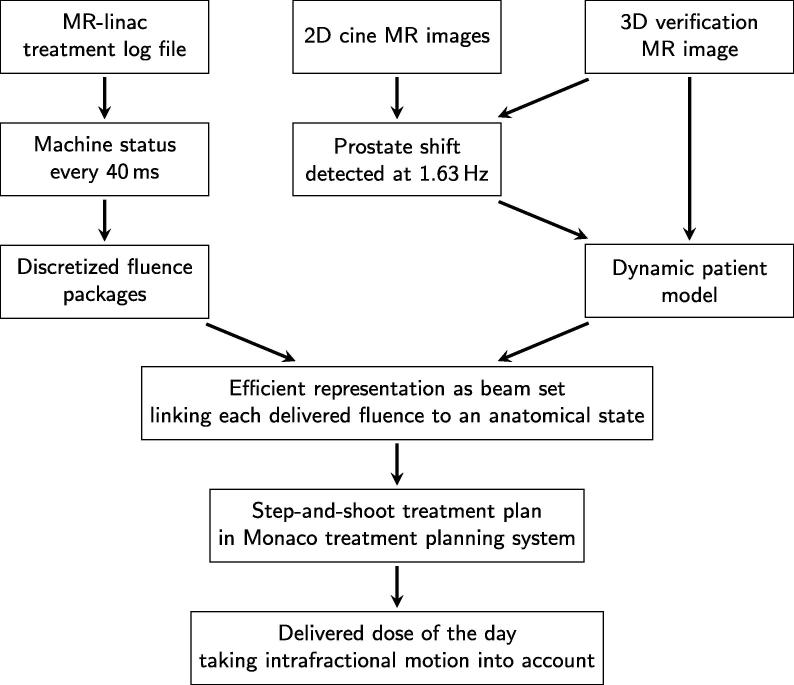
Flow chart describing the dose reconstruction workflow.

The deployed dose reconstruction workflow evolved from a set of tools that were originally developed to validate real-time dose accumulation methods [26]. It aims at calculating the delivered dose by combining continuous information about the treatment machine status with a dynamic model of the patient anatomy (see Fig. 1).

The Unity MR-linac status was recorded every 40 ms. The machine log files included the treatment beam state (on or off), delivered monitor units, gantry angle and the multileaf collimator’s leaf and jaw positions. Each log entry was interpreted as a *discretized fluence package* that was delivered to the specific patient anatomy encountered at the time of delivery. In this study, we modeled the changing anatomy by rigidly shifting the entire verification image according to the previously extracted prostate motion trajectory [3], [27]. Machine log file and motion trajectory were synchronized based on their time stamps and the trajectory was linearly interpolated to match the time points of the MR-linac log file. Afterwards, the isocenter of each fluence package was shifted to represent the relative offset between anatomy and treatment beam. In order to reduce the number of fluence packages to a workable amount, those with similar isocenters, beam angles and multileaf collimator shapes were grouped together. Fluence packages were deemed similar using binning sizes of 1.19 mm (the pixel size of the 2D MR images) for the isocenter positions, 1° for the beam angle and 0.5 mm difference in a single leaf position for the different aperture shapes.

The resulting beam set was imported into the Elekta Monaco treatment planning system, research version 5.59.02, via a software tool based on a vendor-provided C++ research interface. The beam set was encoded as a single step-and-shoot IMRT treatment plan with multiple isocenters. This approach allowed us to efficiently calculate the delivered dose on the verification image using a clinically validated dose calculation engine and MR-linac machine model including the 1.5 T magnetic field [28], [29]. The dose was calculated as dose-to-medium on a grid of 0.25 × 0.25 × 0.25 cm^3^ with a statistical uncertainty of 1%.

### Evaluation

We analyzed data from the first five prostate cancer patients treated with the Unity MR-linac at our institute. Of the 100 delivered fractions, we were able to obtain the 3D verification image, 2D cine MR images and treatment log file in 90 cases. For these fractions, we analyzed the magnitude and characteristics of the prostate motion. We report both the one-off shift of the prostate between the acquisition of the 3D verification image and 2D cine MR imaging as well as the motion trajectory of the primary CTV during 2D cine MR imaging.

Additionally, we compared the delivered dose calculated by our dose reconstruction to the planned dose distribution. The simple patient model used in this study is able to accurately resolve the dose to the prostate and nearby regions [27]. More distant structures may move differentially to the monitored target. For this reason, we only evaluated the dose delivered to the primary CTV and the near-maximum dose to the adjacent organs-at-risk. We report the minimum dose received by 98% of the CTV (CTV $D_{98\%}$), CTV $D_{50\%}$, bladder $D_{3\%}$ and rectum $D_{3\%}$. To facilitate this analysis, these structures were contoured on the verification images by an expert physician.

In this study, we treated each delivered fraction independently and did not use any interfractional dose accumulation. However, we do scale all dose changes by a factor of 20 in order to facilitate an easier comparison to the clinical intent of delivering 60 Gy in 20 fractions.

## Results

We successfully reconstructed the delivered dose of the day in 89 out of 90 fractions. In a single case, the template matching algorithm was unable to accurately localize the prostate in the 2D cine MR images. During this fraction, the patient involuntarily urinated during treatment. The urine traversing the urethra appeared brightly in the 2D cine MR images. This large change in image intensity greatly reduced the accuracy of the template matching algorithm.

**Fig. 2:**
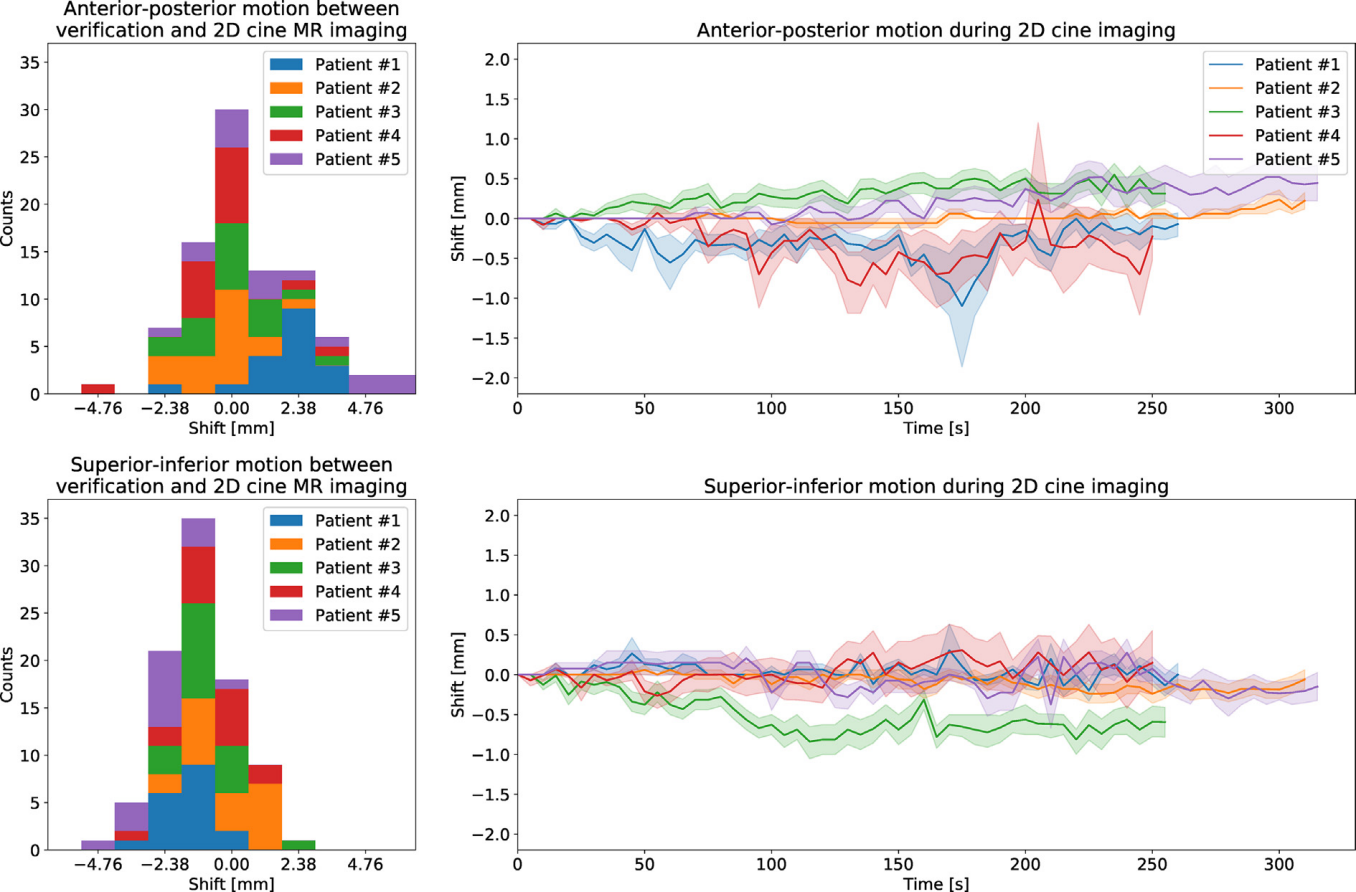
Analysis of the observed prostate motion. The histograms on the left present the shift occurring between the acquisition of the 3D verification images and the beginning of 2D cine MR imaging. The trajectories on the right denote the mean shift at each time point during 2D cine imaging. The shaded area denotes one standard deviation in either direction across all fractions per patient.

**Fig. 3:**
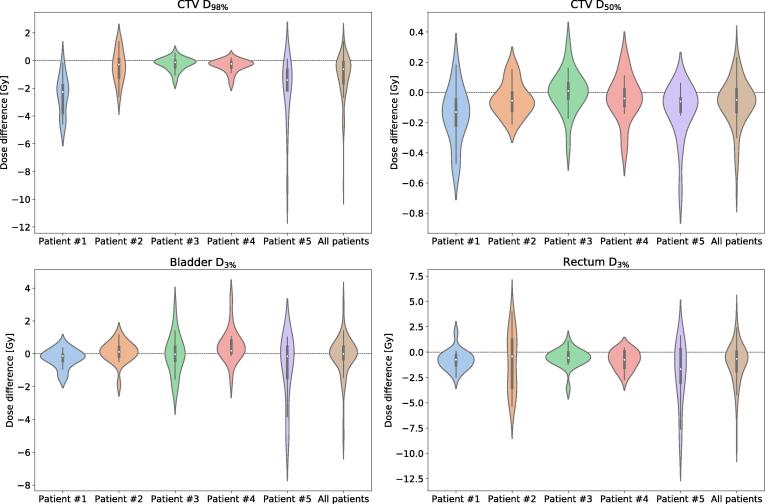
Violin plots presenting the difference between the delivered and planned dose, quantified via four dose-volume metrics. The width of each violin is governed by the probability density of the data points. Additionally, small box plots are included in each violin. The box marks the first and third quartile, while the white dot denotes the median. The whiskers represent the 1.5 interquartile range.

Between the acquisition of the 3D verification image and the beginning of 2D cine MR imaging, 6.4 min ± 2.3 min (mean ± standard deviation) passed. In this time, the CTV moved by 0.5 mm ± 1.3 mm in posterior direction and 1.1 mm ± 2.0 mm in inferior direction (see Fig. 2). Afterwards, patients were surveyed with 2D cine MR imaging for 5.5 min ± 0.8 min. During this time, in which the radiation beam was turned on, an additional shift of 0.0 mm ± 0.8 mm along the anterior-posterior axis and 0.1 mm ± 0.9 mm in inferior direction was detected. Extrapolating the dose changes in each case to all 20 fractions, the mean CTV $D_{98\%}$ and CTV $D_{50\%}$ dose-volume metrics decreased by 1.1 Gy ± 1.6 Gy and 0.1 Gy ± 0.2 Gy, respectively (see Fig. 3). Bladder $D_{3\%}$ did not change (0.0 Gy ± 1.2 Gy), while rectum $D_{3\%}$ decreased by 1.0 Gy ± 2.0 Gy.

**Fig. 4:**
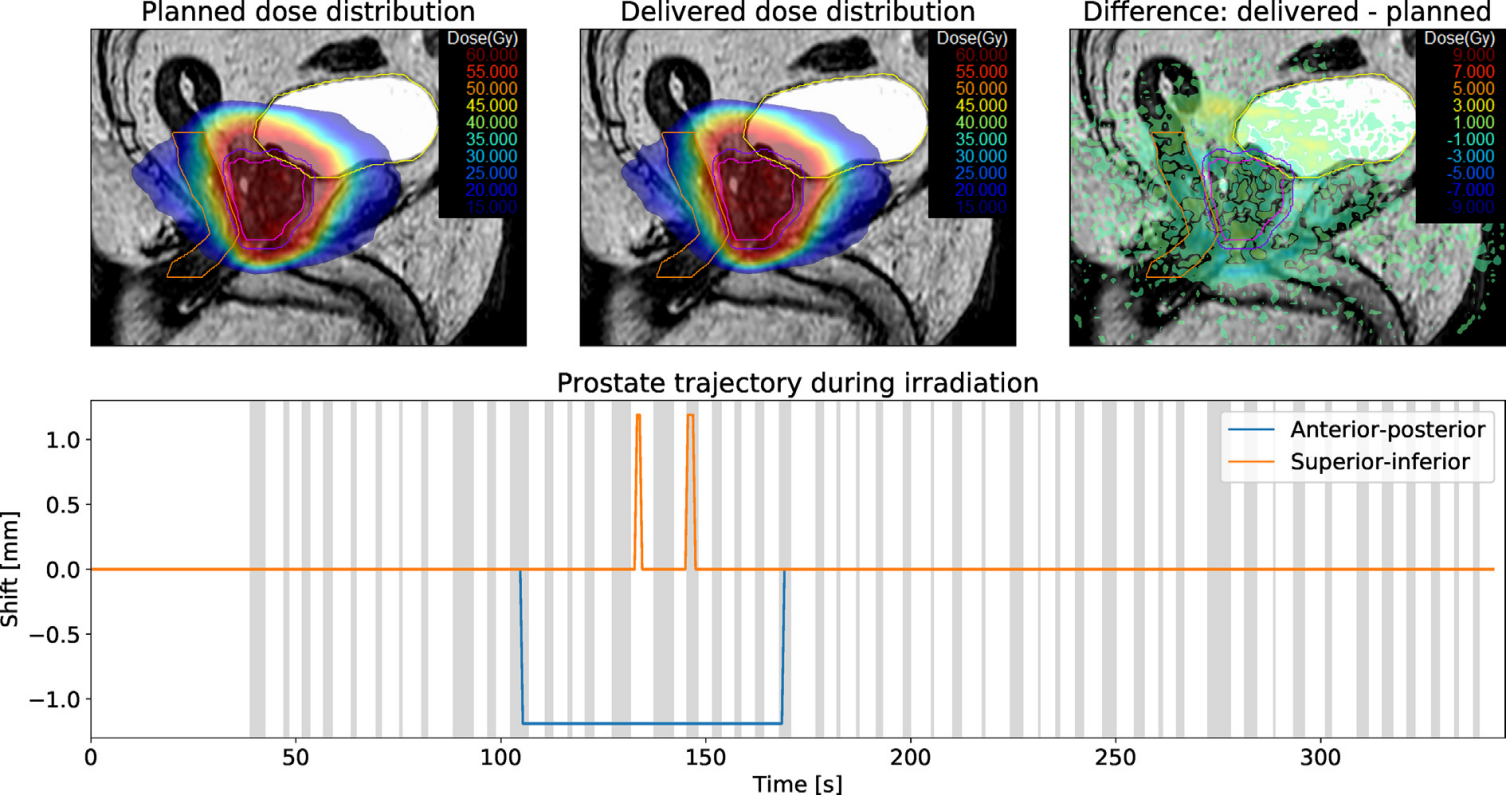
Summary of patient #2, fraction 19. The top row shows the planned dose, reconstructed delivered dose and dose difference overlaid over a sagittal MR-image. The measured CTV motion trajectory is shown in the bottom row. Areas shaded in gray denote times during which the treatment beam was turned on.

Both the observed prostate motion and its dosimetric impact were small in the majority of cases. Detected CTV position and dosimetric evaluation of a typical fraction are shown in Fig. 4. In the presented case, the CTV did not shift by more than a single pixel (1.19 mm). Consequently, the delivered dose distribution was very similar to the planned dose and all investigated dose-volume metrics remained constant.

**Fig. 5:**
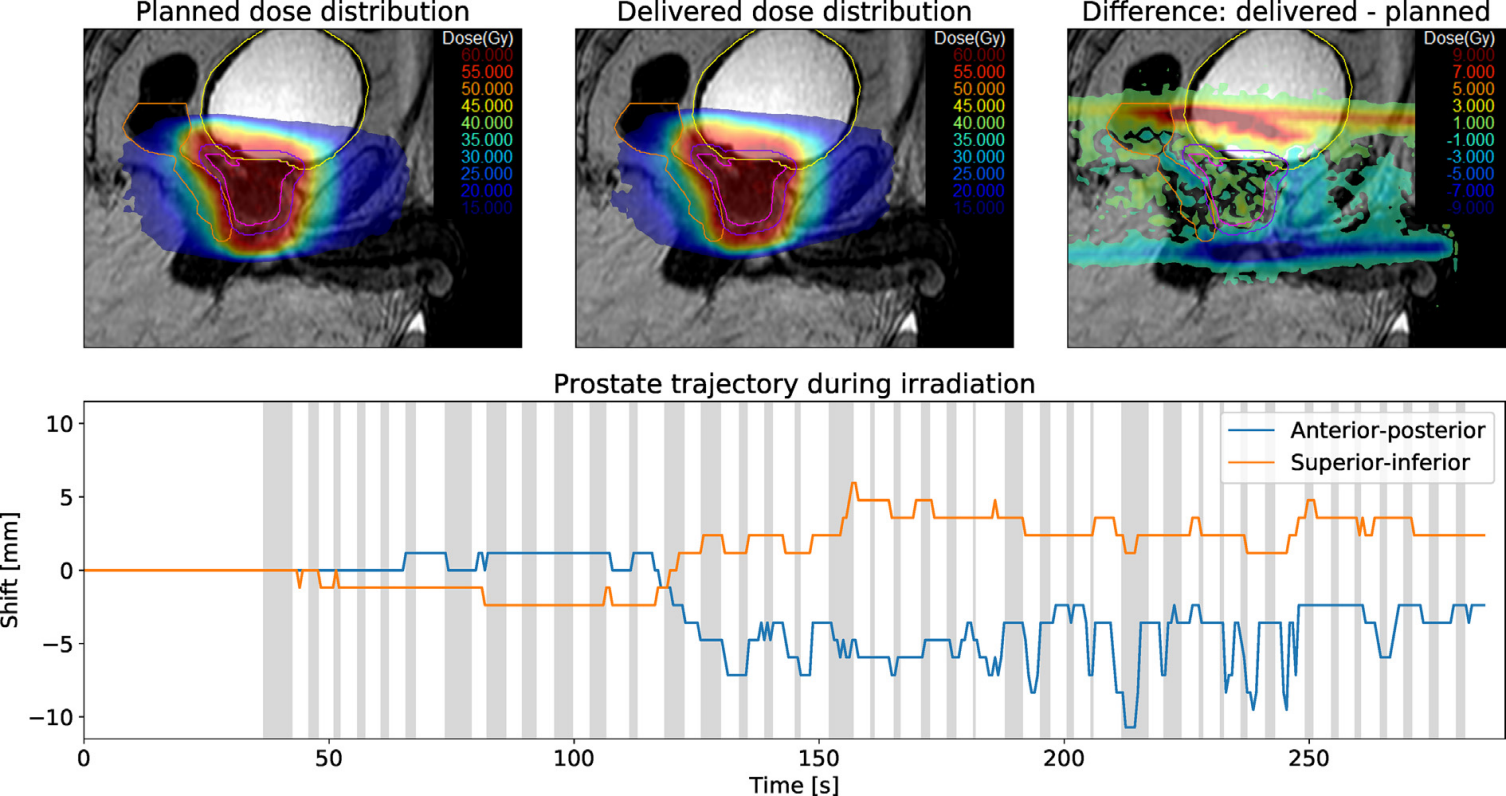
Summary of patient #4, fraction 17. The top row shows the planned dose, reconstructed delivered dose and dose difference overlaid over a sagittal MR-image. The measured CTV motion trajectory is shown in the bottom row. Areas shaded in gray denote times during which the treatment beam was turned on.

However, in a few cases the CTV moved substantially after the final treatment plan was approved on the verification image. Fig. 5 depicts a fraction with large intrafractional motion. Initially, the prostate did not shift between the acquisition of verification image and beginning of 2D cine MR imaging. After the first two treatment beams were delivered, air bubbles passed through the rectum. Over the course of the delivery, the CTV’s mean shift was 2.6 mm in anterior direction and 1.1 mm in superior direction. Despite the large observed motion, the delivered CTV dose changed only slightly. Bladder $D_{3\%}$ and rectum $D_{3\%}$ increased by 0.8 Gy and 0.4 Gy, respectively.

**Fig. 6:**
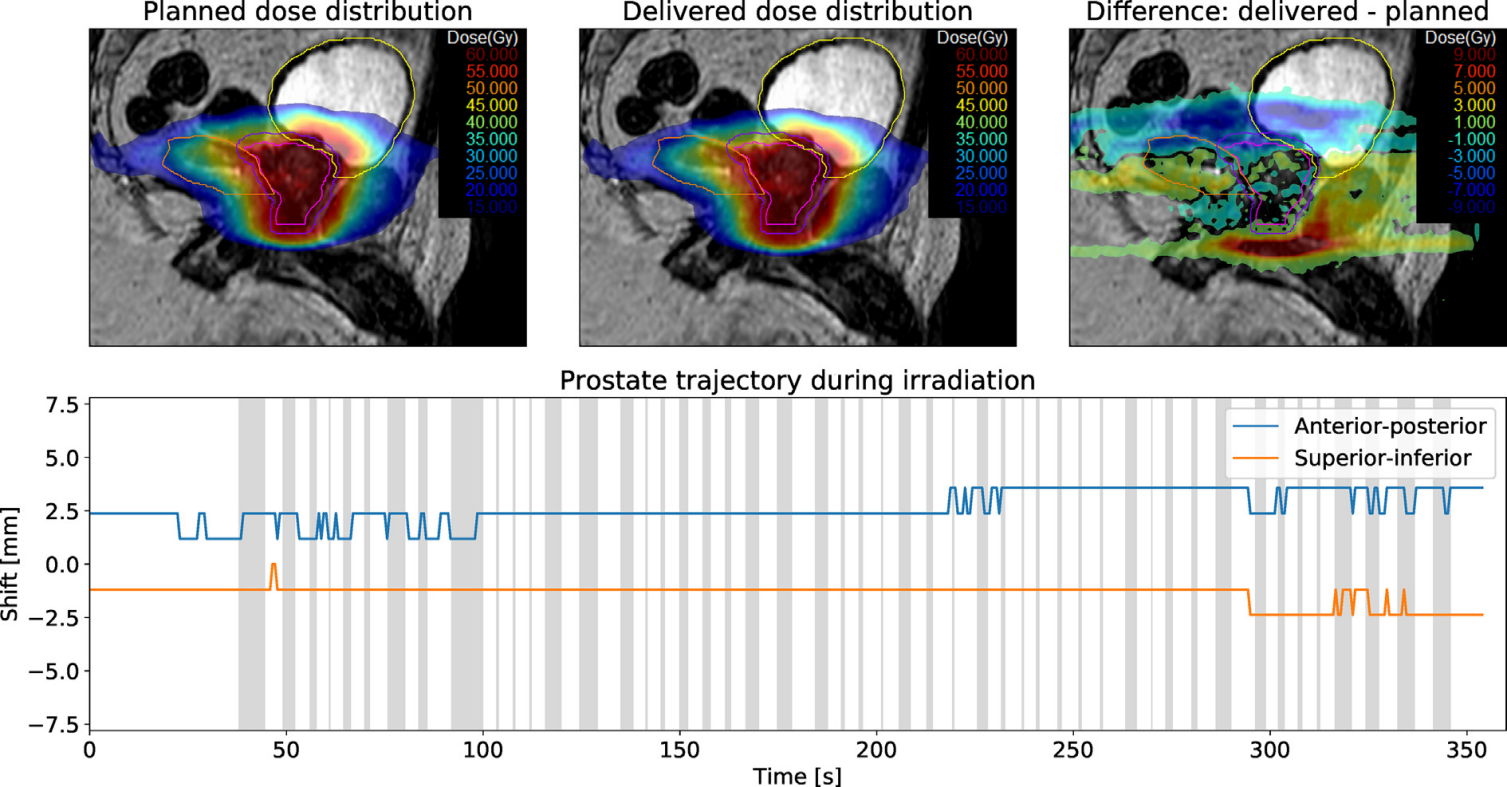
Summary of patient #1, fraction 5. The top row shows the planned dose, reconstructed delivered dose and dose difference overlaid over a sagittal MR-image. The measured CTV motion trajectory is shown in the bottom row. Areas shaded in gray denote times during which the treatment beam was turned on.

Another case with large intrafractional prostate motion is presented in Fig. 6. At the beginning of 2D cine MR imaging the prostate had already moved by 2.38 mm in posterior and 1.19 mm in inferior direction relative to the verification image. While it remained stable during treatment delivery, the offset affects dose coverage of the CTV. Extrapolated to 20 fractions, CTV $D_{98\%}$ decreased by 4.6 Gy and CTV $D_{50\%}$ decreased by 0.2 Gy. Simultaneously, bladder $D_{3\%}$ decreased by 1.5 Gy.

## Discussion

In this study, we have presented an end-to-end pipeline to harness the on-board imaging capabilities of an MR-linac to reconstruct the delivered radiotherapy dose. By processing 3D MR images, 2D cine MR images and treatment machine log files, we were able to measure the intrafractional motion and determine its dosimetric impact in 89 prostate radiotherapy fractions. Contrary to previous dose reconstruction methods, our workflow requires neither the implantation of fiducial or electromagnetic markers nor extra imaging time associated with increased patient discomfort and radiation exposure [3], [30]. In most cases, we found the observed anatomical changes and their effect on the dose distribution to be small and most likely clinically irrelevant. However, in a few fractions large intrafractional motion caused the dose distribution to substantially deviate from the intended plan. Anatomical changes resulted in underdosage of the CTV or additional exposure of the rectum and bladder. Considering the full radiotherapy treatment is delivered over 20 fractions, these deviations arguably only have a negligible effect on clinical outcomes. However, with an increased interest in hypofractionated and stereotactic radiotherapy, dose distortions in a single fraction may become more impactful and important to monitor [31].

Our presented workflow deploys a simple template matching algorithm to detect rigid shifts in prostate position in 2D cine MR images acquired during treatment. The changing patient anatomy is modeled by rigidly shifting a 3D MR image acquired prior to treatment delivery. This patient model is not able to resolve rotations, deformations and density changes affecting the radiation beam’s attenuation. Furthermore, the 2D cine MR sequence used in our clinical workflow consists of three orthogonal imaging planes. As our pipeline only processes the sagittal slice, this imposes an artificial handicap on both the imaging frequency and the accuracy of the template-matching algorithm caused by saturation band artifacts. Nonetheless, we believe that our approach is able to accurately derive the delivered dose to the CTV and adjacent parts of the bladder and rectum.

In theory, the presented workflow can be extended to account for rotations by adapting the template-matching algorithm to detect these and rigidly rotate the planning 3D MR image during dose calculation [32]. A more sophisticated anatomical representation is needed to apply the dose reconstruction workflow to cancer sites that are affected by strong deformations. 2D MR images, as used in this study, are unable to provide a continuous 3D model of the patient’s anatomy on their own. In the future, fast 3D MR acquisition and reconstruction techniques or dynamic patient models may be able to provide a full volumetric representation of the patient over the course of radiation delivery [33], [34], [35]. Owing to the modular nature of our presented workflow, such 3D information could be seamlessly integrated into our dose reconstruction tool in the future. However, use of a dynamically updated patient anatomy requires reliable methods to deformably map the dose delivered at each time point to a reference image [36].

The ability of MR-guided treatment machines to continuously survey the patient can also be used to guide real-time adaptive radiotherapy, such as beam gating or multileaf collimator tracking [37], [15], [38]. Dose reconstruction may be used to assure the correct functioning of these techniques or evaluate their effect on the delivered dose [39]. All relevant information needed to describe these techniques, such as the multileaf collimator’s aperture shapes and the treatment beam status, is already being recorded in the MR-linac log files and can be processed by our developed pipeline.

We have presented a dose reconstruction workflow based on data routinely acquired during MR-guided radiotherapy. We have demonstrated its ability to reliably, non-invasively and automatically reconstruct the delivered dose in 89 prostate radiotherapy fractions. As more and more imaging information becomes available, we envision this workflow to be extended to other cancer sites and ultimately to enter widespread clinical use.

## Declaration of Competing Interest

Michel Moreau and Spencer Marshall are employed by Elekta AB.

## References

[1] Khan F.M., Gibbons J.P. (2014). Khan’s the physics of radiation therapy.

[2] Langen K., Jones D. (2001). Organ motion and its management. Int J Rad Oncol Biol Phys.

[3] Langen K.M., Lu W., Willoughby T.R. (2009). Dosimetric effect of prostate motion during helical tomotherapy. Int J Rad Oncol Biol Phys.

[4] Ehrbar S., Jöhl A., Tartas A. (2017). ITV, mid-ventilation, gating or couch tracking–A comparison of respiratory motion-management techniques based on 4D dose calculations. Radiother Oncol.

[5] Bentzen S.M., Constine L.S., Deasy J.O. (2010). Quantitative analyses of normal tissue effects in the clinic (QUANTEC): an introduction to the scientific issues. Int J Rad Oncol Biol Phys.

[6] Mutic S., Dempsey J.F. (2014). The ViewRay system: magnetic resonance–guided and controlled radiotherapy.

[7] Lagendijk J.J., Raaymakers B.W., Van Vulpen M. (2014). The magnetic resonance imaging–linac system.

[8] Keall P.J., Barton M., Crozier S. (2014). The Australian magnetic resonance imaging–linac program.

[9] Fallone B.G. (2014). The rotating biplanar linac–magnetic resonance imaging system.

[10] Acharya S., Fischer-Valuck B.W., Kashani R. (2016). Online magnetic resonance image guided adaptive radiation therapy: first clinical applications. Int J Rad Oncol Biol Phys.

[11] Raaymakers B., Jürgenliemk-Schulz I., Bol G. (2017). First patients treated with a 1.5 T MRI-Linac: clinical proof of concept of a high-precision, high-field MRI guided radiotherapy treatment. Phys Med Biol.

[12] Acharya S., Fischer-Valuck B.W., Mazur T.R. (2016). Magnetic resonance image guided radiation therapy for external beam accelerated partial-breast irradiation: evaluation of delivered dose and intrafractional cavity motion. Int J Rad Oncol Biol Phys.

[13] de Koste J.R.v.S, Palacios M.A., Bruynzeel A.M., Slotman B.J., Senan S., Lagerwaard F.J. (2018). MR-guided gated stereotactic radiation therapy delivery for lung, adrenal, and pancreatic tumors: a geometric analysis. Int J Rad Oncol Biol Phys.

[14] Thomas D.H., Santhanam A., Kishan A.U. (2018). Initial clinical observations of intra-and interfractional motion variation in MR-guided lung SBRT. Brit J Radiol.

[15] Green O.L., Rankine L.J., Cai B. (2018). First clinical implementation of real-time, real anatomy tracking and radiation beam control. Med Phys.

[16] PRISM trial protocol. https://clinicaltrials.gov/ct2/show/NCT03658525; 2018. Accessed online on the 11th July 2019.

[17] Dunlop A., McQuaid D., Nill S. (2015). Comparison of CT number calibration techniques for CBCT-based dose calculation. Strahlenther Onkol.

[18] Winkel D., Bol G.H., Kroon P.S. (2019). Adaptive radiotherapy: the Elekta Unity MR-linac concept. Clin Transl Rad Oncol.

[19] Ahunbay E.E., Peng C., Chen G.P. (2008). An on-line replanning scheme for interfractional variations. Med Phys.

[20] Balter J.M., Sandler H.M., Lam K., Bree R.L., Lichter A.S., Ten Haken R.K. (1995). Measurement of prostate movement over the course of routine radiotherapy using implanted markers. Int J Rad Oncol Biol Phys.

[21] Bernstein M.A., King K.F., Zhou X.J. (2004). Handbook of MRI pulse sequences.

[22] Bjerre T., Crijns S., af Rosenschöld P.M. (2013). Three-dimensional MRI-linac intra-fraction guidance using multiple orthogonal cine-MRI planes. Phys Med Biol.

[23] Brunelli R. (2009). Template matching techniques in computer vision: theory and practice.

[24] Maes F., Collignon A., Vandermeulen D., Marchal G., Suetens P. (1997). Multimodality image registration by maximization of mutual information. IEEE Trans Med Imag.

[25] Briechle K, Hanebeck UD. Template matching using fast normalized cross correlation. In: Optical Pattern Recognition XII; vol. 4387. International Society for Optics and Photonics; 2001. pp. 95–102.

[26] Kamerling C.P., Fast M.F., Ziegenhein P., Menten M.J., Nill S., Oelfke U. (2017). Online dose reconstruction for tracked volumetric arc therapy: real-time implementation and offline quality assurance for prostate SBRT. Med Phys.

[27] Poulsen P.R., Schmidt M.L., Keall P., Worm E.S., Fledelius W., Hoffmann L. (2012). A method of dose reconstruction for moving targets compatible with dynamic treatments. Med Phys.

[28] Hissoiny S., Raaijmakers A., Ozell B., Després P., Raaymakers B.W. (2011). Fast dose calculation in magnetic fields with GPUMCD. Phys Med Biol.

[29] Ahmad S.B., Sarfehnia A., Paudel M.R. (2016). Evaluation of a commercial MRI Linac based Monte Carlo dose calculation algorithm with geant 4. Med Phys.

[30] Adamson J., Wu Q., Yan D. (2011). Dosimetric effect of intrafraction motion and residual setup error for hypofractionated prostate intensity-modulated radiotherapy with online cone beam computed tomography image guidance. Int J Rad Oncol Biol Phys.

[31] Benjamin L.C., Tree A.C., Dearnaley D.P. (2017). The role of hypofractionated radiotherapy in prostate cancer. Curr Oncol Rep.

[32] Goshtasby A. (1985). Template matching in rotated images. IEEE Trans Pattern Anal Mach Intell.

[33] Huttinga NR, van den Berg CA, Luijten PR, Sbrizzi A. Model-based reconstruction of non-rigid 3D motion-fields from minimal k-space data: MR-MOTUS. arXiv:190205776; 2019.

[34] McClelland J.R., Hawkes D.J., Schaeffter T., King A.P. (2013). Respiratory motion models: a review. Med Image Anal.

[35] Stemkens B., Tijssen R.H., De Senneville B.D., Lagendijk J.J., Van den Berg C.A. (2016). Image-driven, model-based 3D abdominal motion estimation for MR-guided radiotherapy. Phys Med Biol.

[36] Samavati N., Velec M., Brock K.K. (2016). Effect of deformable registration uncertainty on lung SBRT dose accumulation. Med Phys.

[37] Menten M.J., Fast M.F., Nill S., Kamerling C.P., McDonald F., Oelfke U. (2016). Lung stereotactic body radiotherapy with an MR-linac–Quantifying the impact of the magnetic field and real-time tumor tracking. Radiother Oncol.

[38] Glitzner M., Woodhead P.L., Borman P.T., Lagendijk J.J., Raaymakers B.W. (2019). MLC-tracking performance on the Elekta unity MRI-linac. Phys Med Biol.

[39] Colvill E., Poulsen P.R., Booth J., O’Brien R., Ng J., Keall P. (2014). DMLC tracking and gating can improve dose coverage for prostate VMAT. Med Phys.

