# Supplementary material for: Automatic reconstruction of the delivered dose of the day using MR-linac treatment log files and online MR imaging
Source: Radiother Oncol. 2020 Apr;145:88–94. doi: 10.1016/j.radonc.2019.12.010 (PMC7191265; doi:10.1016/j.radonc.2019.12.010)
Supplement: Supplementary file 1 [file mmc1.pdf]

Supplementary material

Table 1: Mandatory treatment planning constraints used in the PRISM protocol.

Structure	Dose constraint
Primary PTV	$D_{98\%} > 57.0\,\mathrm{Gy}$
	$D_{50\%} > 59.4\,\mathrm{Gy}$
	$D_{50\%} < 60.6\,\mathrm{Gy}$
Secondary PTV	$D_{98\%} > 46.17\,\mathrm{Gy}$
	$D_{50\%} > 48.6\,\mathrm{Gy}$
Bladder	$V_{52.7\,\mathrm{Gy}} < 50\%$
	$V_{56.7\,\mathrm{Gy}} < 35\%$
	$V_{60.8\,\mathrm{Gy}} < 25\%$
Rectum	$V_{40.5\,\mathrm{Gy}} < 60\%$
	$V_{48.6\,\mathrm{Gy}} < 50\%$
	$V_{52.7\,\mathrm{Gy}} < 30\%$
	$V_{56.8\,\mathrm{Gy}} < 15\%$
	$V_{60.8\,\mathrm{Gy}} < 5\%$
Bowel	$V_{36.5\,\mathrm{Gy}} < 158\,\mathrm{cm}^3$
	$V_{40.5\,\mathrm{Gy}} < 110\,\mathrm{cm}^3$
	$V_{44.6\,\mathrm{Gy}} < 28\,\mathrm{cm}^3$
	$V_{48.7\,\mathrm{Gy}} < 6\,\mathrm{cm}^3$
	$V_{52.7\,\mathrm{Gy}} < 0.01\,\mathrm{cm}^3$
Individual femoral joints	$V_{40.5\,\mathrm{Gy}} < 50\%$
Penile bulb	$V_{40.5\,\mathrm{Gy}} < 50\%$

Table 2: Sequence parameters of the balanced steady-state free precession sequence used to acquired 2D cine MR images on the 1.5 T Unity MR-linac.

Sequence parameter	Value
Repetition time	3.64 ms
Echo time	1.82 ms
Flip angle	40°
Pixel bandwidth	1076 Hz
Field of view	$400 \times 400\,\mathrm{mm}^2$
Acquisition matrix size	132×132
Acquired voxel size	$3.0 \times 3.0 \times 5.0\,\mathrm{mm}^3$
Reconstruction matrix size	336×336
SENSE factor	3.0 (RL)
Reconstructed voxel size	$1.19 \times 1.19 \times 5.00\,\mathrm{mm}^3$

Table 3: Sequence parameters of the sequence used to acquire T2-weighted 3D verification images on the 1.5 T Unity MR-linac.

Sequence parameter	Value
Repetition time	1535 ms
Echo time	278 ms
Flip angle	90°
Pixel bandwidth	740 Hz
Field of view	$400 \times 400 \times 300\,\text{mm}^3$
Acquisition matrix size	$268 \times 268 \times 300$
Acquired voxel size	$1.5 \times 1.5 \times 2.0\,\text{mm}^3$
Reconstruction matrix size	$480 \times 480 \times 300$
Reconstructed voxel size	$0.83 \times 0.83 \times 2.00\,\text{mm}^3$
Slice spacing	1 mm
SENSE factor	3.6 (RL)
Partial Fourier (Halfscan Y)	0.62
